# Substrate Reduction Therapy for Krabbe Disease: Exploring the Repurposing of the Antibiotic D-Cycloserine

**DOI:** 10.3389/fped.2021.807973

**Published:** 2022-01-18

**Authors:** Steven M. LeVine, Sheila Tsau

**Affiliations:** Department of Molecular and Integrative Physiology, University of Kansas Medical Center, Kansas City, KS, United States

**Keywords:** D-cycloserine, hemopoietic stem cell transplantation, Krabbe disease, NMDA receptor, psychosine, serine palmitoyltransferase, substrate reduction therapy, twitcher mice

## Abstract

Krabbe disease is a lysosomal storage disease that is caused by a deficiency in galactosylceramidase. Infantile onset disease is the most common presentation, which includes progressive neurological deterioration with corresponding demyelination, development of globoid cells, astrocyte gliosis, etc. Hemopoietic stem cell transplantation (HSCT) is a disease modifying therapy, but this intervention is insufficient with many patients still experiencing developmental delays and progressive deterioration. Preclinical studies have used animal models, e.g., twitcher mice, to test different experimental therapies resulting in developments that have led to progressive improvements in the therapeutic impact. Some recent advances have been in the areas of gene therapy and substrate reduction therapy (SRT), as well as using these in combination with HSCT. Unfortunately, new experimental approaches have encountered obstacles which have impeded the translation of novel therapies to human patients. In an effort to identify a safe adjunct therapy, D-cycloserine was tested in preliminary studies in twitcher mice. When administered as a standalone therapy, D-cycloserine was shown to lengthen the lifespan of twitcher mice in a small but significant manner. D-Cycloserine is an FDA approved antibiotic used for drug resistant tuberculosis. It also acts as a partial agonist of the NMDA receptor, which has led to numerous human studies for a range of neuropsychiatric and neurological conditions. In addition, D-cycloserine may inhibit serine palmitoyltransferase (SPT), which catalyzes the rate-limiting step in sphingolipid production. The enantiomer, L-cycloserine, is a much more potent inhibitor of SPT than D-cycloserine. Previously, L-cycloserine was found to act as an effective SRT agent in twitcher mice as both a standalone therapy and as part of combination therapies. L-Cycloserine is not approved for human use, and its potent inhibitory properties may limit its ability to maintain a level of partial inactivation of SPT that is also safe. In theory, D-cycloserine would encompass a much broader dosage range to achieve a safe degree of partial inhibition of SPT, which increases the likelihood it could advance to human studies in patients with Krabbe disease. Furthermore, additional properties of D-cycloserine raise the possibility of other therapeutic mechanisms that could be exploited for the treatment of this disease.

## Introduction

Krabbe disease is an autosomal recessive, lysosomal storage disease caused by mutations in the *GALC* gene leading to a deficiency of galactosylceramidase (1, 2). This enzyme normally cleaves galactose from the substrates galactosylceramide and psychosine, resulting in ceramide and sphingosine, respectively. Galactosylceramide is a major sphingolipid that is enriched within myelin (3, 4). If galactosylceramidase is deficient, then instead of galactose being removed during recycling in the lysosome, the fatty acid moiety is removed from galactosylceramide by acid ceramidase (5). This results in the production of psychosine (a.k.a. galactosylsphingosine). Psychosine is normally kept at very low levels due to toxic properties, but if galactosylceramidase is deficient, then the levels of psychosine dramatically increase (6–8). The accumulation of psychosine is thought to be the main mediator of pathology in Krabbe disease, e.g., oligodendrocyte death, demyelination, and disruption of a variety of cellular functions (2, 9, 10).

The most common presentation of the disease is infantile onset. Initial symptoms which can include irritability, crying, hypersensitivities to touch, stiffness of limbs, etc., appear by 3 months and are followed by progressive deterioration leading to a fatal outcome by 2 or 3 years (2, 11). Other forms of the disease include late infantile onset, juvenile onset, and adult onset disease (2, 12). Some patients with later forms of disease may have residual galactosylceramidase activity (13), but residual enzyme levels do not predict the disease course (14). Adding the measurement of psychosine levels, e.g., within dried blood spots, may help with differentiating the different onset forms of disease, which is relevant for identifying possible candidates for the therapeutic intervention of hemopoietic stem cell transplantation (HSCT) (15, 16). Although HSCT can be used as a treatment of the infantile forms of disease, it is frequently inadequate with developmental delays and disease progression often still occurring in patients that received this intervention (17–19). Due to this shortcoming, there have been numerous preclinical studies directed at identifying other therapeutic approaches for this disease.

Here we provide a brief review of various experimental therapies. In addition, we introduce a drug, previously approved by the FDA for other conditions, that is a candidate for further evaluation. Developing a new adjunct treatment for patients with Krabbe disease, particularly one that could be used in combination with HSCT, would help to slow disease activity and move the field closer to halting disease activity altogether.

## Experimental Therapies

A variety of therapeutic approaches have been evaluated for Krabbe disease. Pre-clinical, *in vivo* testing most often utilizes twitcher mice, which have a mutation that occurred naturally in their *GALC* gene and display many parallels with the condition in humans (20). Twitcher mice have a progressive disease course with clinical presentations that include tremor, ataxia, weakness, weight loss and premature death (20, 21). Pathological findings include demyelination in both the CNS and PNS, astrocyte gliosis, microglial gliosis, and accumulation of globoid cells (20). Various experimental therapies include HSCT, gene therapy, enzyme replacement therapy (ERT), chaperone therapy, anti-inflammatory treatments, and substrate reduction therapy (SRT) (10, 21). In preclinical studies, no single therapy has resulted in a complete blockage of the pathogenic process, with many tests of individual treatments obtaining only mild slowing of disease in twitcher mice. All experimental approaches have revealed obstacles that have impaired the effectiveness of the treatment. The obstacles include achieving insufficient levels of the corrected enzyme, the blood-brain barrier limiting the delivery of corrected enzyme or viral vector to the CNS, the development of various adverse side effects including some of which are severe, etc. Despite these obstacles, there has been steady progress toward the goal of developing therapies that have increased efficacy, e.g., lengthening of lifespan (22–25).

## Hemopoietic Stem Cell Transplantation

HSCT is the only disease modifying therapy with a published track record of use in humans with Krabbe disease, but this approach has shortcomings. HSCT (inclusive of bone marrow transplantation) was shown in 1984 to increase the lifespan of twitcher mice (26). A key mechanism accounting for this benefit is the delivery of functional enzyme by the progeny of transplanted cells (25, 27, 28). Additional mechanisms include reducing inflammation (29).

In human patients with Krabbe disease, HSCT has yielded mixed results, with the treatment often more successful in patients with late onset disease rather than early onset disease (17–19, 30–32). Treated infantile patients can experience developmental delays and still succumb to the disease (17–19, 32). Improved outcomes are obtained when HSCT is performed prior to the onset of symptoms and preferably within the first 30 days of life (18). Making a correct diagnosis and then initiating treatment within this time frame is a substantial challenge (33, 34). Although asymptomatic patients can be identified by neonatal screening, the great majority of states do not perform this testing (35), which creates a significant obstacle to implementing treatment within the recommended timeframe. Even if treatment is initiated within 30 days, the outcome following HSCT is still uncertain with many patients undergoing developmental delays and possibly an early death (18, 35). Thus, HSCT is insufficient for many patients. A second therapy, particularly one that works synergistically with HSCT, could substantially improve therapeutic outcomes.

## Combination Therapy

Preclinical testing of other experimental therapies has shown varying degrees of success with some of the best outcomes achieved by utilizing a combination of therapies. For instance, HSCT has been combined with SRT and/or gene therapy. The combinations consistently yield better outcomes than the matched individual therapies (22, 24, 25, 36). Other approaches such as ERT, chaperone therapy, and anti-inflammatory therapy also have the potential to contribute as part of combination or standalone therapies.

Although a combination intervention using gene therapy is intriguing, recent findings have raised some potential issues with this approach. Specifically, experimental gene therapy using an AAV2/9 vector delivered systemically has been shown to lead to hepatocellular carcinoma when used in combination with HSCT or both HSCT and SRT (24). The explanation postulated for the high incidence of cancer was due to integration at key sites, e.g., *Rian* locus (24). In addition, the ionizing irradiation used for the pretreatment conditioning regimen for HSCT causes DNA mutations, and the SRT agent L-cycloserine lowers ceramide levels which normally would have an antiproliferative effect (24, 37). Interestingly, neither HSCT nor L-cycloserine by themselves have been found to cause hepatocellular carcinoma in treated mice (24). Other pretreatment regimens for HSCT (e.g., busulfan, cyclophosphamide) can result in serious adverse effects such as infertility and graft vs. host disease (25).

Besides having the potential to cause cancer, when gene therapy is used on isolated hematopoietic stem and progenitor cells to induce high *GALC* expression levels, it results in toxicity and impairs transplantation (25, 38). To get around this difficulty, the incorporation of a regulatory system to control expression, or incorporation of a promoter to target expression to differentiated progeny, is necessary (38). Thus, gene therapy approaches have current limitations that may impede their use in combination therapy with HSCT.

To avoid combination therapy using HSCT, gene therapy using increased dosages of viral vectors has been explored. Administration of high doses of viral vectors gave a much better response than lower doses, including those used by other gene therapy protocols (25). Furthermore, no overt signs of toxicity were noted, but the results (e.g., lifespan) still fell short of those with combination therapy (25).

Since inflammatory processes have been implicated in promoting disease activity, anti-inflammatory agents have been investigated for possible therapeutic values. In fact, some studies indicate that the effects of HSCT are due to anti-inflammatory properties (29, 39). Ibudilast is an anti-inflammatory drug that inhibits several phosphodiesterases and migration inhibitory factor, which may have a role in inflammation including that mediated by microglia (40–42). Twitcher mice treated with ibudilast may have shown lessened signs of CNS pathology (43). Additional anti-inflammatory drugs (i.e., indomethacin, minocycline, ibuprofen) were found to lengthen the lifespan of transgenic mice with low levels of galactosylceramidase activity (44). However, combining gene therapy with indomethacin failed to improve the outcome in twitcher mice compared to gene therapy alone (25), suggesting that a more aggressive anti-inflammatory approach is needed or that inflammation does not significantly drive pathology.

A more promising approach for combination therapy may include SRT. Currently, SRT agents (e.g., miglustat, eliglustat) are used as a treatment for Gaucher disease type 1 (45), which like Krabbe disease, is a sphingolipidosis. The combination of SRT with ERT has been explored in preliminary studies on some patients, including type 3 Gaucher (46–48).

## Substrate Reduction Therapy Using L-Cycloserine

Myelin is a principal pathological site of Krabbe disease. Myelin has a very high concentration of galactosylceramide (3, 4), which is a sphingolipid digested by galactosylceramidase. As mentioned previously, due to the deficiency of galactosylceramidase in Krabbe disease, galactosylceramide cannot undergo normal digestion to ceramide. Instead, it gets converted by acid ceramidase to psychosine (5), the toxic compound that accumulates and mediates pathology in Krabbe disease (9).

The rate-limiting step of sphingolipid synthesis is catalyzed by serine palmitoyltransferase (SPT) (a.k.a., 3-ketodyhydrosphingosine synthase) (49, 50). L-Cycloserine crosses the blood-brain barrier and irreversibly inhibits SPT. Since the production of galactosylceramide is downstream from SPT, L-cycloserine administration causes a reduction of galactosylceramide synthesis (51–53). In addition, in twitcher mice treated with L-cycloserine, psychosine levels are reduced (22), which is probably a consequence of the lowered production of galactosylceramide since psychosine is derived from the digestion of galactosylceramide by acid ceramidase (5). This lower level of psychosine likely accounts for the lengthened lifespan of twitcher mice treated with L-cycloserine (22, 54, 55). In additional work, when L-cycloserine was combined with HSCT, there was a synergistic beneficial effect (36), and when HSCT and SRT were combined with gene therapy, the beneficial result was enhanced substantially further (22, 24).

Although these results are both intriguing and impressive, L-cycloserine is not approved for human use; and at elevated levels, it can cause toxicity, which may be due to its potent irreversible inhibition of SPT and/or other mechanisms (56, 57). Since sphingolipids perform a variety of essential functions across many tissues, complete inhibition of SPT cannot be tolerated (58, 59), and some levels of partial inhibition could have adverse effects that would likely be dose and time dependent. Thus, in theory, there is a relatively narrow dosage regimen for L-cycloserine that would achieve an acceptable level of partial inhibition of SPT that can reduce pathology without causing toxicity.

## Substrate Reduction Therapy Targeting Ceramide Galactosyltransferase

In a search for safer and perhaps more effective SRT agents, downstream enzymes have been targeted. The gene *UGT8* encodes for ceramide galactosyltransferase (CGT), which catalyzes the final step for galactosylceramide synthesis. Following a search of 30,000 compounds for their ability to inhibit CGT, a thienopyridine was identified and further optimized, resulting in a drug (compound 19) that is an efficacious *in vivo* inhibitor of CGT that is both orally bioavailable and enters the CNS (60). *In vivo* testing in normal mice revealed that this compound was able to inhibit the production of both galactosylceramide and sulfatide in the brain and kidney (60). Additional *in vivo* studies that examine the therapeutic efficacy in mouse models of Krabbe disease and/or metachromatic leukodystrophy are anticipated.

In another study, after starting with a small screen of lipid inhibitors against CGT, a brain penetrant compound was ultimately developed that was quite selective, i.e., not readily inhibiting other enzymes that utilize related substrates (61). This compound (S202) was found to significantly prolong the lifespan of twitcher mice, i.e., from a median of 39.5 up to 63.5 days with treatment starting on postnatal day (PND) 15 (61). In addition, it lowered brain galactosylceramide and psychosine levels, particularly when treatment was started early, i.e., PND 3 (61). These impressive results were dampened by the finding that vacuoles developed in both the white and gray matter of normal mice, even at relatively low doses of the drug (61). In addition, extensive histopathological alterations were observed in the testes (61). The findings of vacuolation, particularly in gray matter, raised the possibility that CGT is performing a previously unknown role, perhaps within astrocytes and/or within other cell types (61). Due to the potential for serious adverse effects, the inhibition of CGT may not be a suitable target to achieve SRT.

## Substrate Reduction Therapy Targeting Acid Ceramidase

In a note-worthy study by Li et al. (5), it was demonstrated that psychosine is derived from a catabolic reaction, the removal of the fatty acid from galactosylceramide by acid ceramidase, rather than an anabolic reaction between galactose and sphingosine to generate psychosine (galactosylsphingosine) as previously proposed (62). Since psychosine is considered to be the toxic metabolite in Krabbe disease, this finding raised the possibility that psychosine production could be reduced by partial inhibition of acid ceramidase. The acid ceramidase inhibitor carmofur was able to lower psychosine levels in fibroblasts from a patient with Krabbe disease and in the brains of twitcher mice (5). However, treatment with carmofur did not extend the lifespan of twitcher mice (63); rather, it was only effective in twitcher mice that were also heterozygous for acid ceramidase deficiency (5). Unfortunately, human cancer patients treated with carmofur can have drug related side effects that include leukoencephalopathy, liver dysfunction, neuropathy, skin rash, and diarrhea (64–67). The potential side effect of leukoencephalopathy is worrisome since the pathological changes ongoing in Krabbe disease may make the patient more susceptible to this adverse effect.

Studies to develop an acid ceramidase inhibitor with optimal properties are actively being pursued. Toward this goal, the development of benzoxazolone carboxamides led to piperidine 22 m, which can cross into the CNS and can be delivered by the oral route (68). This inhibitor reduced both psychosine levels in the brains of twitcher mice and glucosylsphingosine levels in a mouse model of Gaucher disease (68). Additional compounds that inhibit acid ceramidase are also being developed (69). Besides examining the effects of these drugs on psychosine levels, assessing their ability to lessen the overall course of disease, e.g., increase lifespan, in mouse models as well as determining the profile of toxic effects will provide useful information for assessing whether a drug should advance toward human studies.

## Substrate Reduction Therapy: Revisiting Inhibition of Serine Palmitoyltransferase

In an effort to identify a safe SRT agent for Krabbe disease, our attention turned to D-cycloserine, which is the enantiomer of L-cycloserine. Unlike L-cycloserine, D-cycloserine is approved for human use, receiving FDA approval in 1964 (70). D-Cycloserine is used as an antibiotic (i.e., Seromycin) administered by oral daily doses lasting 2 years for the treatment of drug resistant tuberculosis, and it is also used for some urinary tract infections. Both enantiomers inhibit SPT, i.e., in microsomes prepared from mouse brains, but the D enantiomer is 100x less efficient than the L enantiomer (51). Greater inhibition by L-cycloserine than by D-cycloserine was also observed for bacterial SPT (57). A reduced efficiency for the D enantiomer should provide a broader drug concentration range to achieve partial enzyme inhibition. In contrast, the concentration range for the L enantiomer to achieve an equivalent level of partial inhibition would be much narrower and more difficult to achieve. In fact, unlike L-cycloserine, brain microsomal SPT was not inhibited after a single dose of D-cycloserine administered by i.p. injection to mice (51), which should not be surprising given the short duration between administration and sacrifice and the slower inhibition of SPT by D-cycloserine (57). Thus, the broader dosage range for D-cycloserine, in theory, should confer greater safety by keeping the extent of enzyme inhibition below levels that would cause toxicity.

**Figure 1:**
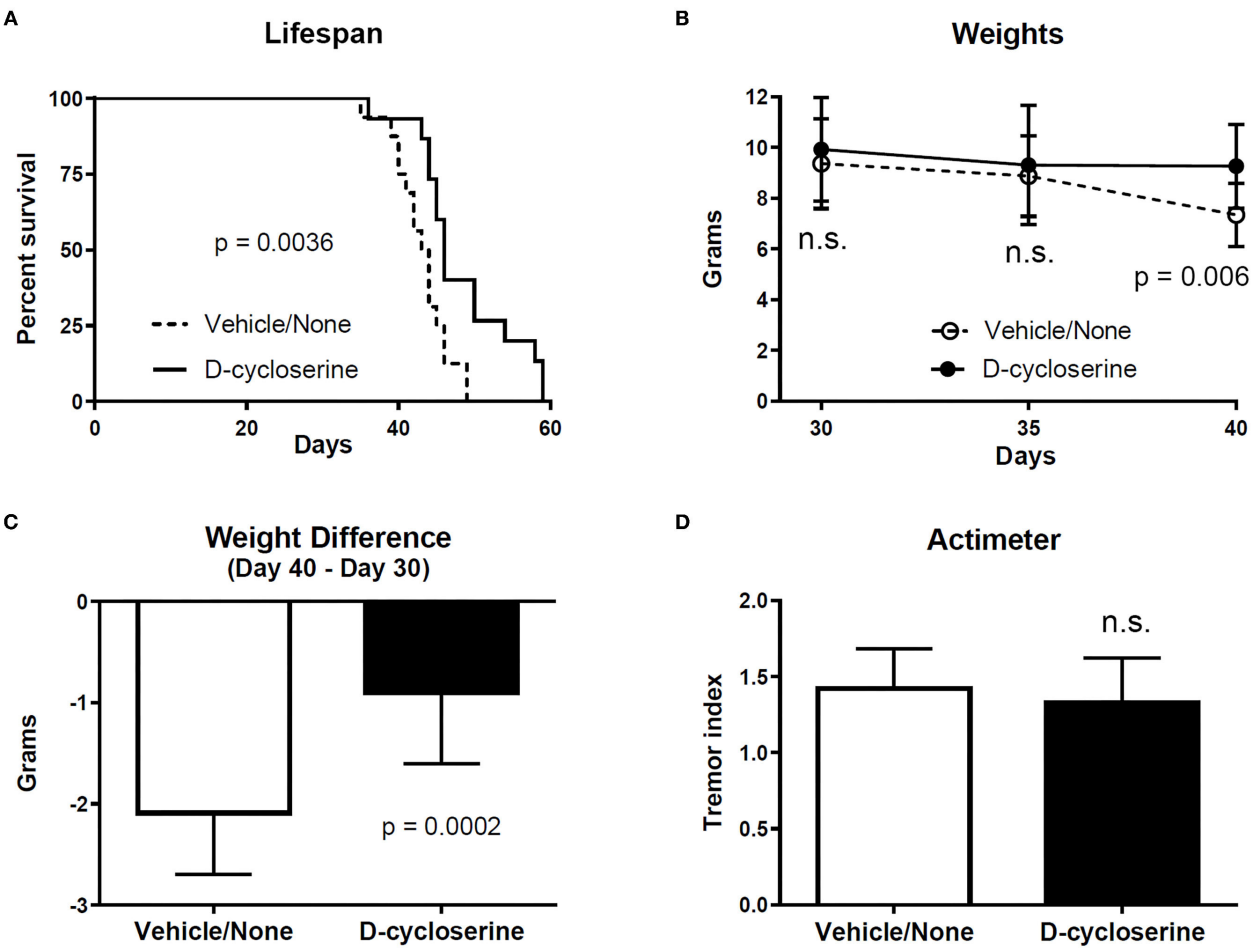
D-cycloserine slows some parameters of disease in twitcher mice. **(A)** The lifespan of twitcher mice receiving D-cycloserine was significantly longer (*p* = 0.0036) than twitcher mice that received vehicle or no treatment (these latter two groups were combined). Log-rank (Mantel-Cox) test. Since the data for males was not statistically different from females within each group, data for males and females were combined within the D-cycloserine group (male *n* = 9; female *n* = 6) as well as within the vehicle/no treatment group (male *n* = 8; female *n* = 8). **(B)** Weights (mean ± S.D.) were not different between twitcher mice given D-cycloserine and twitcher mice given vehicle/no treatment at PND 30 (*n* = 16 and *n* = 15, respectively) and PND 35 (*n* = 15 and *n* =15, respectively) but were significantly greater (*p* = 0.006) in twitcher mice given D-cycloserine at PND 40 (*n* = 14 and *n* = 14, respectively) (one mouse in the vehicle group was at PND 39). Student *t*-test, two-tailed, corrected for multiple comparisons. **(C)** Weight loss (weight at PND 40 minus weight at PND 30; mean with S.D., although one mouse in the vehicle group was at PND 39) was significantly less (*p* = 0.0002) for twitcher mice receiving D-cycloserine (*n* = 14) vs. twitcher mice receiving vehicle/no treatment (*n* = 14) (Mann Whitney, two-tailed test). **(D)** Twitching severity (i.e., tremor index, mean with S.D.), as measured by actimeter (23) at PND 34–36, was statistically not significant (n.s.) in D-cycloserine treated twitcher mice (*n* = 14) vs. vehicle/no treatment twitcher mice (*n* = 15) (two-tailed Student *t*-test, p ≤ 0.05 as significant).

In preliminary studies, we found that D-cycloserine prolongs the lifespan of twitcher mice (Figure 1A). In addition, it resulted in greater body weight in twitcher mice at PND 40, but not PND 30 or 35 (Figure 1B), and it lessened weight loss in twitcher mice (Figure 1C). Twitching severity, as measured by an actimeter (23), was not statistically different between treated vs. non-treated groups of twitcher mice (Figure 1D), suggesting that this measure is better for detecting larger effect sizes (5, 23, 29). The dosage of D-cycloserine used for these early findings was 200 mg/kg *via* daily i.p. injections starting on PND 4 or 5. In contrast, in previous studies on twitcher mice the dosage of L-cycloserine was kept at or below 75 mg/kg administered every other day (54), or at or below 50 mg/kg 3x per week (22).

Additional studies examining D-cycloserine in twitcher mice, and in other models of Krabbe disease, are warranted. Besides ascertaining if these preliminary findings can be replicated, determining the effects of D-cycloserine on the development of pathology and psychosine accumulation, e.g., within twitcher mice, would be informative. In addition, it would be particularly interesting to examine the combined effect of D-cycloserine with HSCT to see if the outcome improved compared to that for either treatment alone.

More refined dosing regimen studies are also required. The blood half-life of D-cycloserine in mice is only 23 min (71). This raises the possibility that more frequent dosing could give rise to a greater benefit in twitcher mice. The half-lives of D-cycloserine in the monkey and human are substantially longer, i.e., 7.75 h (71) and 8–12 h (72), respectively. Given this longer half-life, it is likely that a considerably lower dosage than that used for mice would be effective in humans. Being able to use a lower dosage may also provide a greater safety buffer.

Combining D-cycloserine with pyridoxine (a.k.a., vitamin B6) may improve the outcome following treatment. D-Cycloserine forms a stable complex with pyridoxal 5′-phosphate (PLP), the active form of vitamin B6 (a.k.a., pyridoxine); therefore, it can potentially impair enzymes that utilize PLP (73–76). Since many PLP requiring enzymes are used by the brain, e.g., for the generation and degradation of neurotransmitters (77), co-administration of D-cycloserine with pyridoxine may offset some potential adverse consequences (78, 79). However, SPT also utilizes PLP as a co-factor (50) and D-cycloserine targets the co-factor resulting in inhibition (57). Thus, if the therapeutic effect of D-cycloserine is *via* the inhibition of SPT, then offsetting adverse effects on other PLP utilizing enzymes by administering pyridoxine may be difficult to achieve without negating the desired inhibition of SPT.

## Other Putative Mechanisms for D-Cycloserine

Previously, it has been noted that lipopolysaccharide (LPS) can accelerate disease activity in twitcher mice, and the effects are mediated, at least in part, by proinflammatory cytokines (80, 81). In addition, both a febrile infection and an influenza A infection have been found to precipitate the presentation of late-infantile and juvenile onset Krabbe disease, respectively (82, 83). Thus, infections leading to proinflammatory conditions might amplify disease activity. Since D-cycloserine is an antibiotic, it is possible that the disease slowing effects of this treatment observed in twitcher mice were due to its antibiotic properties. However, antibiotic effects in mice may be diminished due to the short drug half-life in this species (71).

D-Cycloserine also has anti-inflammatory effects, at least in macrophages. In RAW macrophages stimulated with LPS, D-cycloserine reduced the phosphorylation of extracellular signal-regulated kinases (84). In these cells, D-cycloserine also inhibited the expression of both inducible nitric oxide synthase and cyclooxygenase 2, and the corresponding production of nitric oxide and downstream production of prostaglandin E2, respectively (84). D-Cycloserine also inhibited the expression of IL-1 beta and IL-6 and reduced the phosphorylation of inhibitory kappa B-alpha while increasing the non-phosphorylated form in macrophages activated by LPS (84), which would decrease the activation of NF-κB and lower the production of proinflammatory cytokines. These anti-inflammatory properties could account for the therapeutic effects of D-cycloserine in twitcher mice.

Besides having antibiotic and anti-inflammatory properties as well as inhibiting SPT, D-cycloserine is a partial agonist of the NMDA receptor by interacting with the binding site for the co-agonist glycine (72). As such, numerous studies have examined the ability of D-cycloserine to treat anxiety disorders, including those in children (85, 86). It is possible that some of the therapeutic activity of D-cycloserine in twitcher mice is *via* this agonist property. How activation of the NMDA receptor would improve survival is unclear but could involve various molecular pathways such as increasing phosphorylation of protein kinase A and cAMP response element binding protein (87). Additionally, unlike its action in LPS-induced macrophage cells, where it downregulated phosphorylation of ERK to reduce inflammation (84), D-cycloserine may increase phosphorylation of ERK when acting as an NMDA agonist (87). Of note, in a patient with adult onset Krabbe disease, glutamate levels in the corticospinal tract and frontal brain were decreased compared to controls (88), and the partial agonist property of D-cycloserine on the glutamate NMDA receptor could help offset this deficiency. Furthermore, D-cycloserine was found to have protective effects in models of seizures, traumatic brain injury, and Parkinson's disease (89–93), which would support the possibility that it could also lessen disease activity in twitcher mice by a mechanism other than inhibiting SPT.

## D-Cycloserine–Safety Considerations

D-Cycloserine has been tested in children and adolescents for the treatment of anxiety disorders, and both infants and fetuses have been exposed to the drug during nursing and pregnancy, respectively (86, 94–96). Although short-term, low dosages are considered relatively safe in adults (72), its safety in young individuals and/or at prolonged higher doses is less clear. D-Cycloserine has the potential for a range of adverse effects including a variety of neuropsychiatric presentations, GI issues, rare cardiac effects, rash, etc., which would be expected to be more pronounced with higher and/or prolonged dosages (72, 97). Furthermore, the safety of this drug specifically within patients with Krabbe disease is not known. Given the biochemical and pathological changes in Krabbe disease, there are some areas that warrant heightened attention relative to safety in this patient population, even if the drug is administered at a low dose. For instance, since the CNS is a main site of pathology in Krabbe disease, it is possible that CNS adverse effects would be induced at a lower dose, have a greater frequency, or be amplified in affected individuals.

A sensitivity to glutamate has been implicated as a mechanism that promotes disease activity in models of Gaucher disease (98, 99). For instance, partial activation of the NMDA receptor using D-cycloserine worsened disease in a chemically induced model of this disease in mice (98). Gaucher disease is due to mutations in the glucosylceramidase gene (a.k.a., acid beta-glucosidase). This lysosomal storage disease results in defective glucosylceramide and glucosylsphingosine catabolism. Many gangliosides, which are built upon glucosylceramide, are concentrated in neurons. Unlike Gaucher disease, the catabolism of galactosylceramide and psychosine (galactosylsphingosine) in Krabbe disease are disrupted principally within myelin, not neurons. Although a pathogenic role of NMDA receptor activation *via* D-cycloserine would not be as expected for Krabbe disease compared to Gaucher disease, this possibility cannot be excluded.

Since D-cycloserine potentially disrupts PLP-containing enzymes by forming a stable complex with PLP (73–76), the activity of one or more of these enzymes could be affected and function at a deficient level. Co-administering D-cycloserine with pyridoxine may act to offset these effects (78, 79); but whether this would also counter the inhibition of SPT is unclear, and it might be necessary to have a staggered or other type of administration regimen. In fact, it would have been interesting to see if the pathogenic effects attributed to D-cycloserine in a mouse model of Gaucher disease (98) could have been offset by pyridoxine coadministration. It is worth noting that administration of high doses of pyridoxine, with or without D-cycloserine administration, can lead to peripheral neuropathy (100), indicating that a carefully selected dose of pyridoxine is necessary.

Outside the CNS, sphingolipids perform essential roles in several major organs including the gastrointestinal tract and heart (101, 102). Since SPT catalyzes an early step in sphingolipid biosynthesis, it is possible that the normal functions performed by sphingolipids will be disrupted following partial inhibition of this enzyme. Of note, there have been reports of gastrointestinal and cardiac issues with the use of D-cycloserine (72). It is also possible that the toxic effects of D-cycloserine in a mouse model of Gaucher disease (98) were due to an excessive reduction of sphingolipids, especially if some recycling was prevented by the chemically induced deficiency of glucosylceramidase.

The list of potential areas of concern discussed above is not complete. Thus, besides additional preclinical studies examining therapeutic mechanisms and efficacy, further studies addressing the safety of D-cycloserine are necessary prior to testing in humans with Krabbe disease.

## Hypothesis

We hypothesize that D-cycloserine, an FDA approved drug for tuberculosis, can be repurposed to ameliorate disease activity in Krabbe disease. Three pieces of information help establish the foundation for this hypothesis: (1) preliminary findings indicate that D-cycloserine prolongs the life of twitcher mice, (2) D-cycloserine inhibits SPT (51, 57), which is the rate-limiting step for sphingolipid production (49, 50), and (3) reductions of SPT activity result in reduced production of galactosylceramide and psychosine, which are the substrates for the deficient enzyme galactosylceramidase in Krabbe disease (103).

It is possible that D-cycloserine acts independently of SPT inhibition to help ameliorate Krabbe disease by having anti-inflammatory effects, being a partial agonist of the NMDA receptor, by functioning as an antibiotic, or through other mechanisms. But for D-cycloserine to advance to testing in human patients with Krabbe disease, it will need to be relatively safe, and the safety of D-cycloserine likely will be correlated with the dosage. Since the drug has a half-life substantially longer in humans than mice (71, 72), a corresponding effective dosage in humans is expected to be much lower than that used in twitcher mice. In addition, the drug is soluble in water and deliverable by the oral route, which should make delivering multiple doses per day easier in the human infant than for very young mice. Administering multiple doses per day to human patients should allow for a relatively lower dosage to be effective compared to the single daily dose used in twitcher mice that is rapidly cleared.

If D-cycloserine can be used as an SRT, or other form of therapy, for Krabbe disease, then it could be implemented in various ways. For instance, in patients with milder presentations of the disease, i.e., adult onset forms of the disease, D-cycloserine could be used as a standalone therapy. These patients may not be candidates for HSCT, and if D-cycloserine results in a small diminution of disease activity, it could be enough to offset the slower disease progression in late-onset Krabbe disease. Additionally, D-cycloserine could be used in a combination therapy for infantile onset disease, which is the most common form of disease that has an aggressive rate of progression and requires HSCT. Interestingly, besides being studied in numerous trials for conditions other than tuberculosis, D-cycloserine has been examined as part of combination therapies in both animal and human studies. For instance, in animals, D-cycloserine has been combined with pioglitazone as a possible treatment for a mouse model of chronic orofacial neuropathic pain (104). In humans, D-cycloserine has been used as part of a combination treatment regimen with lurasidone for bipolar depression or acute suicidal ideation and behavior in bipolar depression (105, 106).

To improve the prospects for a better outcome, HSCT ideally would be performed by 30 days of life, prior to the onset of symptoms (18). However, implementing this intervention within this timeframe can be difficult, especially since most states currently do not require neonatal testing for Krabbe disease. By slowing the disease course, SRT used early, in theory, could extend the window of time for HSCT to impact the disease. In addition, since infantile onset disease is a rapidly progressing condition, time is precious, but HSCT requires time to fully take effect, e.g., healthy donor cells need to engraft, repopulate, and disperse in the body. By slowing the disease, SRT would provide more time for the donor cells to undergo these essential processes, allowing for a greater opportunity to affect disease activity. Furthermore, HSCT by itself often fails to stop the progression of disease. Since SRT should slow disease progression, adding it to HSCT should result in a greater chance that the disease will be substantially ameliorated. In addition, if SRT can improve the outcome following HSCT, then it is likely that more states will opt for neonatal testing, opening the possibility for more patients to receive a disease modifying treatment.

If D-cycloserine is found to function as an SRT for Krabbe disease, then in theory, it could have potential therapeutic value for other lysosomal storage disorders involving sphingolipids. These diseases could affect other galactosyl-sphingolipids (e.g., metachromatic leukodystrophy), glucosyl-sphingolipids (e.g., Gaucher and GM1 gangliosidosis), sphingomyelin (e.g., Niemann Pick), and/or ceramide (e.g., Farber).

## Summary

The therapies available for patients with Krabbe disease are inadequate, with most patients still experiencing a progressive disease course even despite treatment. Although there has been steady progress in preclinical studies toward the development of more efficacious therapies, several promising approaches have been impeded by adverse consequences. In an effort to identify a new adjunct treatment that is also relatively safe, we considered other drug candidates. D-Cycloserine is an FDA approved drug, i.e., for tuberculosis, and has been studied in a large number of clinical trials for other conditions. Preliminary findings indicate that it can lessen the disease course in twitcher mice; thus, D-cycloserine deserves further evaluation to determine its potential to advance to testing in patients with Krabbe disease. The putative mechanism for D-cycloserine would be *via* inhibition of SPT acting as an SRT, although other mechanisms that affect the disease course are possible. Identifying a small molecule such as D-cycloserine that crosses the blood-brain barrier (107), is relatively safe at least at low dosages (72), and lessens the disease process, is rare and represents an opportunity to make rapid advancements toward the goal of developing better therapies.

### Method

Animal experiments were approved by the animal care and use committee of the University of Kansas Medical Center and were in accordance with the guidelines of the National Institutes of Health. Twitcher mice were produced by mating heterozygous mice that were originally provided by Ernesto Bongarzone, Ph.D., University of Illinois at Chicago, Chicago, IL. Mice had access to food (regular chow or breeder chow) and water *ad libitum* and were given access to 93M Nutrigel (Clear-H_2_O, Portland, ME) as the disease advanced. In preparation for drug administration, stock solutions of D-cycloserine (Sigma, St. Louis) were prepared at 100 mg/ml in filtered deionized H_2_O, aliquoted, and frozen at −20°C. A mixture of 1 part stock solution and 4 parts saline was prepared prior to injection. Twitcher mice were given daily i.p. injections of D-cycloserine at 200 mg/kg starting on PND 4 or 5. Additional twitcher mice received injections of saline starting at PND 4 or 5, or no injections.

## Data Availability Statement

The original contributions presented in the study are included in the article, further inquiries can be directed to the corresponding author.

## Ethics Statement

The animal study was reviewed and approved by Animal Care and Use Committee of the University of Kansas Medical Center.

## Author Contributions

SL conceived of the study, wrote the manuscript, guided the experimental studies, and analyzed data. ST carried out experiments, assisted with the design and intellectual components of the study, analyzed data, and assisted with the writing of the manuscript. All authors approved the study.

## Funding

This work was funded by the BioNexus KC 19-1 Patton Trust Research Grant and the University of Kansas School of Medicine Investigator Assistance Award. Core support was provided by the Kansas Intellectual and Developmental Disabilities Research Center (NIH U54 HD 090216).

## Conflict of Interest

SL has received prior funding from ApoPharma, Inc., and has had early interactions with Chiesi. SL is employed by the University of Kansas Medical Center. The remaining author declares that the research was conducted in the absence of any commercial or financial relationships that could be construed as a potential conflict of interest.

## Publisher's Note

All claims expressed in this article are solely those of the authors and do not necessarily represent those of their affiliated organizations, or those of the publisher, the editors and the reviewers. Any product that may be evaluated in this article, or claim that may be made by its manufacturer, is not guaranteed or endorsed by the publisher.
